# Removal of Particulate Matter in a Tubular Wet Electrostatic Precipitator Using a Water Collection Electrode

**DOI:** 10.1100/2012/532354

**Published:** 2012-03-12

**Authors:** Jong-Ho Kim, Hee-Jung Yoo, You-Seong Hwang, Hyeok-Gyu Kim

**Affiliations:** ^1^Department of Environmental Engineering, Hanseo University, Seosan 356-706, Republic of Korea; ^2^Applied Meteorology Research Laboratory, National Institute of Meteorological Research, Seoul 156-720, Republic of Korea

## Abstract

As one of the effective control devices of air pollutants, the wet electrostatic precipitator (ESP) is an effective technique to eliminate acid mist and fine particles that are re-entrained in a collection electrode. However, its collection efficiency can deteriorate, as its operation is subject to water-induced corrosion of the collection electrode. To overcome this drawback, we modified the wet ESP system with the installation of a PVC dust precipitator wherein water is supplied as a replacement of the collection electrode. With this modification, we were able to construct a compact wet ESP with a small specific collection area (SCA, 0.83 m^2^/(m^3^/min)) that can acquire a high collection efficiency of fine particles (99.7%).

## 1. Introduction

As the standard for ambient air quality and emission sources have gradually intensified, numerous strategies have been developed and introduced to air quality management [1–3]. The higher standards have forced industries to use clean energies or to install high-efficiency control devices in order to comply with such regulations [4]. Gas streams released after such treatment systems in diverse source units (e.g., industrial boilers, production processes, etc.) can still contain diverse hazardous air pollutants such as heavy metals, volatile compounds, and high-health-risk polycyclic aromatic hydrocarbons [4–7].

Despite notable advances in control technology, certain fractions of pollutants can still escape and be released into the air. The fine particles released from the treatment stage have light scattering and absorption characteristics that can lead to visibility impairment [8]. Hence, a precipitator used as the main control unit requires a high collection efficiency to remove fine particles. Although a dry electrostatic precipitator shows 99% efficiency in terms of total mass, it generally exhibits a low collection efficiency against fine size fractions compared to the coarse counterparts [9].

The reduced collection efficiency of fine particles (0.1~1.0 *μ*m) is ascribable to the limitation in its inherent charging mechanism and in the re-entrainment of fine particles [10, 11]. In order to overcome such a limitation in the application of the electrostatic precipitator, several attempts were made to improve the collection efficiency of particles (e.g., increase in the size of the precipitator, the use of pulse energization, and so forth [12]). Recently, such techniques as the agglomeration by electric field, acoustic field, and electrospray have also been investigated as alternate approaches [9, 12, 13].

Among the available control techniques, the wet electrostatic precipitator (ESP) is a potent device that can facilitate the efficient collection of fine particles and acid aerosols. The three key components that allow a normal electrostatic precipitation are particle charging, the collection of particles on the collecting electrode, and the removal of the collected particles [14]. In the third stage of the operation, the wet ESP uses water to clean away the collected particles [15]. Because the collected particles are removed by the constant supply of water, the wet ESP can maintain the targeted control efficiency without “re-entrain” and “back corona” which would otherwise lead to a decrease in the collection efficiency [10, 14]. As the wet ESP can be operated in low-temperature conditions, it can also be advantageous in using less treatable gas. The applicability of the wet ESP can be extended further to treat gaseous pollutants, especially soluble gases (e.g., SO_2_, HCl, NH_3_, etc.). In light of the potent role of the wet ESP, one may maximize its usefulness by reducing or eliminating the corrosion induced by water [14].

In order to resolve the drawbacks of the preexisting wet ESP technique, we developed a modified wet ESP in which a PVC tube is installed as a dust precipitator to facilitate the flow of water as an alternate electrode. The inner surface of the PVC was sanded; otherwise it can disrupt water flow due to its imperfections and the surface tension of water. In addition, a spiral feeder was also installed as a water guide on top of the precipitator. All of these modifications were made to optimize the efficiency of the water supply in this modified wet ESP system. For example, the local protrusion of water surface can cause a spark to discharge. The presence of a dry area on the collection electrode can also disrupt the corona discharge to induce a back corona [10, 11].

## 2. Experimental

### 2.1. Instrumental Setup

**Figure 1:**
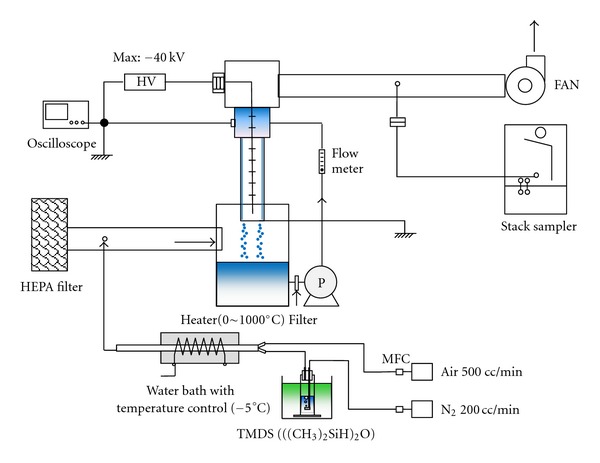
Experimental setup to measure the collection efficiency of the modified wet ESP investigated in this study.

In this study, our modified wet electrostatic precipitation system was built in a clean wind tunnel as shown in Figure 1. The test system consisted of a high-efficiency particulate air (HEPA) filter, a dust generator, and a wet ESP. To provide clean air into the air supply system, the HEPA filter was installed.

**Figure 2:**
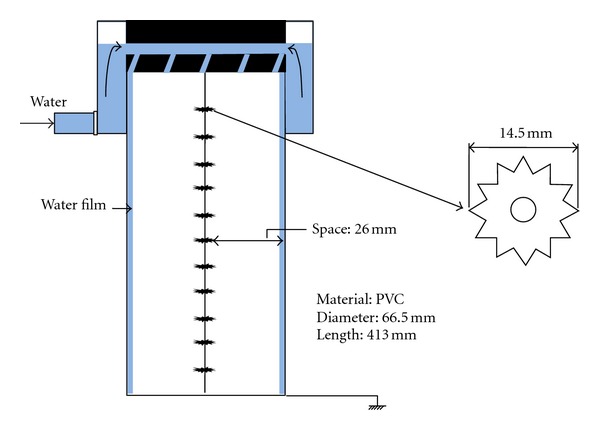
Schematic diagram a modified wet ESP.

To test the efficiency of our improved wet ESP, particles were generated artificially using 1,1,3,3-tetramethyl disiloxane (TMDS, (CH_3_)_2_HSi–O–SiH(CH_3_)_2_, Aldrich). The impinger system with TMDS solution was set in a temperature-controlled water vessel (−5°C). Pure nitrogen gas (0.2 L/min) was fed into the bottle containing TMDS. TMDS was vaporized by bubbling, then mixed with air (0.5 L/min) before entering the electric furnace. The gas-to-particle conversion of TMDS occurred in the electric furnace, which was maintained at 700°C. Gas flows were controlled using mass flow controllers (Kofloc, Model 8300, Japan). As TMDS enters the electric furnace in the form of vapor, it immediately turns into stable oxides. The formation of particles then proceeds in a stepwise manner after nucleation, condensation, and coagulation [9, 16]. A pilot wet ESP was built with a PVC tube of 66.5 mm in diameter and 413 mm in length (Figure 2).

The discharge rod is a stainless steel star-shaped bar that facilitates efficient discharge along the edges as shown in Figure 2. The dust collecting electrode was designed to form a water film on the wall inside the precipitator, and a spiral feeder was installed on the upper side of the precipitator to maintain continuous water supply (Figure 2). In addition, a special sanding finish was applied so that the water was able to flow and spread evenly over the PVC surface.

A DC power supply (ZEPA, Model HM200-40K-SP, Korea) with the capacity of up to −40 kV was used. Because most industrial ESPs are operated with the negative polarity, the performance of ESP was evaluated with high negative DC voltage. The average gas velocity inside the wet ESP was 1.0 m/s which is equivalent to a flow rate of 0.21 Nm^3^/min.

As we intended to examine whether water can be used successfully to replace the dust collecting electrode, water was made to flow evenly on the inner surface of the precipitator. The specifications of the wet ESP in this study are shown in Table 1 along with a common dry ESP for reference [17].

### 2.2. Measurement of Collection Efficiency

Dust collection efficiency was also examined in relation to the power supply by changing their values from the highly negative voltage to the increased levels of −11, −13, and −15 kV. The size distribution and its mass concentration of particles were measured to assess the collection efficiency of the system by using an in-stack cascade impactor (series 220, Sierra instruments, Inc., USA) with isokinetic sampling (EPA method 5). Dp, 50 of each stage for the cascade impactor, is shown in Table 2. The measurement of collection efficiency was carried out more than 7 times at each condition.

 The applied voltage of the wet ESP was measured using a digital oscilloscope (Tektronix, Model TDS 2014B, USA) and a high-voltage probes (Tektronix, Model 6101A, USA). A register of 1 kΩ was inserted to the ground line in series to measure the discharge current in our modified wet ESP. The gas velocity of the inner wet ESP was measured using an anemometer (TSI, Model 9515). The mass removal efficiency (*η*) of the wet ESP was calculated as follows:

*η* = ((concentration of particles with wet ESP off − concentration of particles with wet ESP on)/concentration of particles with wet ESP off).

## 3. Results and Discussion

### 3.1. Generation of Artificial Particles for Collection Efficiency Test

**Figure 3:**
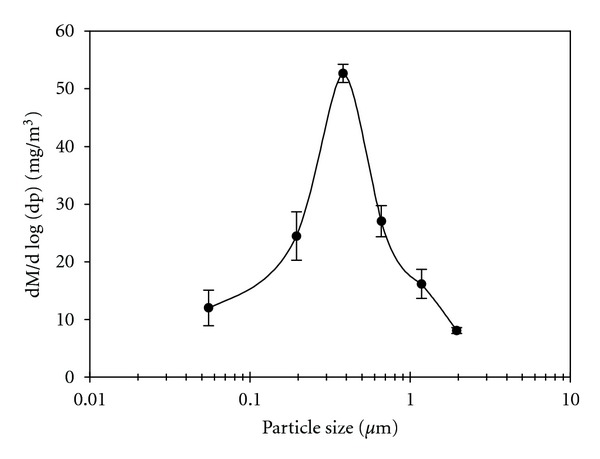
Size distribution of test particles.

As described above, the test particles were generated using the thermal reaction of TMDS. The average concentration of particles was 59.5 mg/m^3^ with a standard deviation of 2.2 and a log-normal distribution of their size fractions (Figure 3). The peak value of fine particles was 0.4 *μ*m, while the particles in the size range of 0.1~1.0 *μ*m were responsible for 65% of the total weight. This size range corresponds to the minimum range to measure the collection efficiency in a normal dry ESP [9, 18, 19].

As stated above, the collection efficiency of particles, especially in the size range that is not easy to remove by the common precipitator, is the most important factor for the operation of the wet ESP. The experimental conditions of our study were thus set to produce the optimum size range of particles.

### 3.2. Characteristics of Voltage-Current for the Wet ESP

The wet ESP was operated with a water feeding rate from 0.5 to 1 L/min, where the inner surface of the PVC maintained was contact at a constant rate. If the water feeding of our setup exceeded 2 L/min, the roughness of the water layer could increase and cause a spark-over condition.

**Figure 4:**
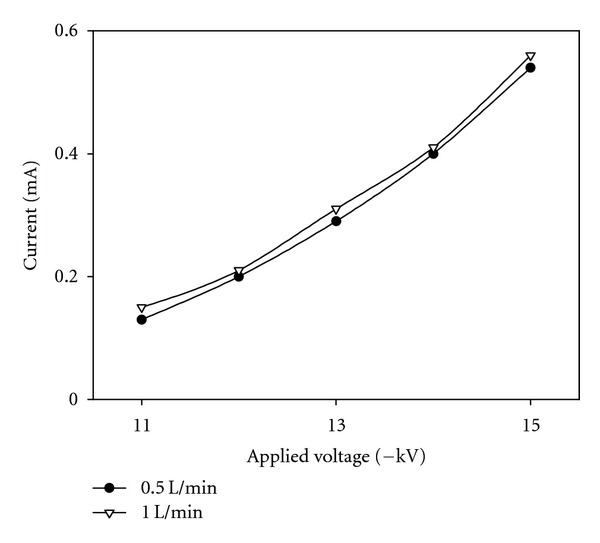
Voltage-current characteristics in the wet ESP.

**Figure 5:**
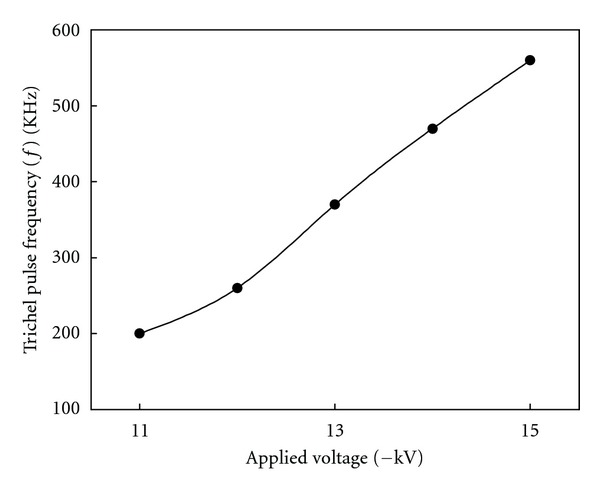
Trichel pulse frequency versus applied voltage in the wet ESP.

Figure 4 shows the current-voltage relationship at the water supply rate of 0.5 and 1 L/min. The electric current began to flow, and the voltage was applied to the wet ESP at −10 kV. At −11 kV, corona discharge began, and the current value rapidly rose in relation to the applied voltage. This is a phenomenon normally found in negative polarity corona [9].  Figure 5 also shows changes in the “Trichel” pulse frequency against the applied voltage. These current pulses correspond to the electron attachment to form negative ions and their migration to the ground electrode by the electric field [20]. The frequency of the Trichel pulse increased with the applied voltage and reached about 550 kHz at −15 kV.

### 3.3. Evaluation of Dust Collection Efficiency

**Figure 6:**
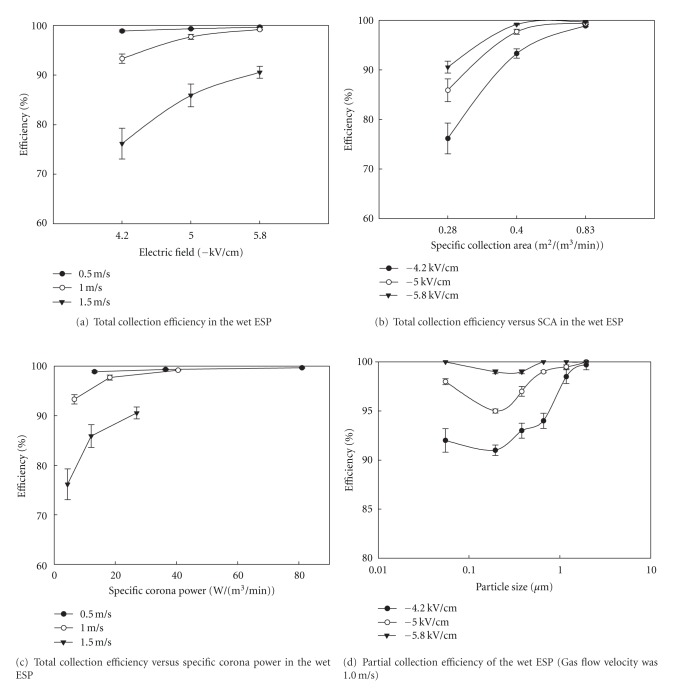
Comparison of particle collection efficiency in terms of the interactive relationship between particle size and key operation variables (i.e., electrical field, SCA, and specific corona power).

Figure 6(a) shows the dust collection efficiency in relation to changes in the electrical field and the flow velocity inside the precipitator. In the experiment at the applied voltage of −11, −13, and −15 kV, the electrical field strengths were calculated as −4.3, −5.0, and −5.8 kV/cm, respectively.

The results confirm that the higher the electrical field strength (and lower the flow velocity inside the precipitator), the higher the dust collection efficiency. The enhanced flow velocity inside the precipitator implies a short retention time of gas which can lead to an increase in the specific collecting area. One of the important design parameters of the ESP is the specific collection area (SCA), which can be sensitively affected by the size of the ESP.  Figure 6(b) depicts the dust collection efficiency in relation to the specific collecting area and electrical field strength; the bigger the specific collecting area and electrical field strength, the higher the dust collection efficiency. The results of this experiment indicate that the SCA value with the minimum (0.28 m^2^/(m^3^/min)) and maximum (0.83 m^2^/(m^3^/min)) led to the dust collection efficiency of 76.2% and 99.7%, respectively. According to the Air Pollution Engineering Manual [21], the SCA value to acquire 99.5% efficiency was estimated as 1.2~1.5 m^2^/(m^3^/min). Although our system was built as a pilot scale, the SCA value of this experiment is relatively small at 0.83 m^2^/(m^3^/min).

Figure 6(c) indicates the relationship between the specific corona power and dust collection efficiency in relation to the change of flow velocity inside the precipitator. Given the same flow velocity inside the precipitator, a higher corona power ratio can lead to the enhancement of dust collection efficiency. Specific corona power (*P*/*Q*) is another designing factor of the ESP, which is the ratio of the corona power (*P* in watt) to the gas flow rate (*Q* in m^3^/min). This index is useful to provide information on the power consumption in ESP [17]. The result of this experiment shows that the specific corona power of 4.4 W/(m^3^/min) is maintained at the lowest dust collection efficiency (76.2%), while 81.0 W/(m^3^/min) is at the highest efficiency (99.7%).

The results of the specific corona power are similar to or higher than the typical value in dry ESP, as shown in Table 1. A similar investigation was carried out by Saiyasitpanich and so forth [15, 22]. Using a collection electrode made out of steel for a wet ESP, with SCA 0.04 m^2^/(m^3^/min) and the specific corona power 35.4 W/(m^3^/min), an 80% collection efficiency was shown. Compared using the same conditions, SCA (0.3 m^2^/(m^3^/min)) shows higher results and the specific corona power (10 W/(m^3^/min)) shows lower results in this study than Saiyasitpanich's results.

Therefore, by considering the relationship between the specific corona power and SCA values simultaneously, one can possibly derive the optimal operation conditions.  Figure 6(d) indicates the partial collection efficiency of each particle size at the electrical field strength of −4.3, −5.0, and −5.8 kV/cm. The collection efficiency reaches the minimum at the particle size range 0.1~0.7 *μ*m. This size range has an intersection between the diffusion and field charging, as observed previously [10, 22].

In addition, after being used in SCA, the washing water must be treated properly (e.g., filtration) because it collects many particles after being used as a collection electrode.

## 4. Conclusion

In this study, a modified wet ESP was built with the installation of a PVC tube so that water can be used to replace a dust collecting electrode. A series of experiments were conducted to measure the dust collection efficiency as a function of the current-voltage, and the results were derived as follows.

1. As a means to improve the performance of the wet ESP, we modified the system with the installation of PVC tube. When the current value and applied voltage were simultaneously raised, its collection efficiency was maximized. The results of our initial test confirmed that water can be used effectively as a replacement of a collection electrode.
2. Unlike the common dry ESP, our modified wet ESP, tested in this experiment, showed a lower SCA value and a high specific corona power. If their interactive relationship is determined optimally, one may select the optimum operational condition for this system.
3. As a result, it can be concluded that a wet ESP equipped with a special sanding finished PVC tube can be used with high efficiency without experiencing the typical defect of a normal wet ESP (i.e., corrosion).

In the future, we are planning to simultaneously compare to performance of a wet ESP and a dry ESP with respect to the particle collection efficiency of both.

## Figures and Tables

**Table 1:** Basic parameters of the modified wet ESP system investigated in this study.

Parameter	Modified ESP	Typical values in dry ESP^a^
Gas velocity (m/s)	1~1.5	1~2
Temperature (°C)	25°C	100~250°C
Electrical field (kV/cm)	~5.8	~7
Specific collecting area (m^2^/(m^3^/min))	0.28~0.83	0.25~2.1
Specific corona power (w/(m^3^/min))	4.4~81.0	1.75~17.5

Typical value taken from Copper and Alley [17].

**Table 2:** Particle size cutoffs of cascade impactor^a^.

Stage no.	F	9	8	7	6	5	4	3	2
Dp, 50 (*μ*m)	—	0.1	0.29	0.47	0.85	1.5	2.4	4.0	10.0

^a^Series 220 in-stack cascade impactor introduction manual, Sierra instruments, Inc.
